# Evaluation and Selection of *Bacillus* Species Based on Enzyme Production, Antimicrobial Activity, and Biofilm Synthesis as Direct-Fed Microbial Candidates for Poultry

**DOI:** 10.3389/fvets.2016.00095

**Published:** 2016-10-20

**Authors:** Juan D. Latorre, Xochitl Hernandez-Velasco, Ross E. Wolfenden, Jose L. Vicente, Amanda D. Wolfenden, Anita Menconi, Lisa R. Bielke, Billy M. Hargis, Guillermo Tellez

**Affiliations:** ^1^Department of Poultry Science, University of Arkansas, Fayetteville, AR, USA; ^2^Facultad de Medicina Veterinaria y Zootecnia, Universidad Nacional Autónoma de México, Ciudad de México, México; ^3^Pacific Vet Group-USA, Inc., Fayetteville, AR, USA

**Keywords:** *Bacillus*, direct-fed microbial, enzyme, antimicrobial, biofilm

## Abstract

Social concern about misuse of antibiotics as growth promoters (AGP) and generation of multidrug-resistant bacteria have restricted the dietary inclusion of antibiotics in livestock feed in several countries. Direct-fed microbials (DFM) are one of the multiple alternatives commonly evaluated as substitutes of AGP. Sporeformer bacteria from the genus *Bacillus* have been extensively investigated because of their extraordinary properties to form highly resistant endospores, produce antimicrobial compounds, and synthesize different exogenous enzymes. The purpose of the present study was to evaluate and select *Bacillus* spp. from environmental and poultry sources as DFM candidates, considering their enzyme production profile, biofilm synthesis capacity, and pathogen-inhibition activity. Thirty-one *Bacillus* isolates were screened for *in vitro* relative enzyme activity of amylase, protease, lipase, and phytase using a selective media for each enzyme, with 3/31 strains selected as superior enzyme producers. These three isolates were identified as *Bacillus subtilis* (1/3), and *Bacillus amyloliquefaciens* (2/3), based on biochemical tests and 16S rRNA sequence analysis. For evaluation of biofilm synthesis, the generation of an adherent crystal violet-stained ring was determined in polypropylene tubes, resulting in 11/31 strains showing a strong biofilm formation. Moreover, all *Bacillus* strains were evaluated for growth inhibition activity against *Salmonella enterica* serovar Enteritidis (26/31), *Escherichia coli* (28/31), and *Clostridioides difficile* (29/31). Additionally, in previous *in vitro* and *in vivo* studies, these selected *Bacillus* strains have shown to be resistant to different biochemical conditions of the gastrointestinal tract of poultry. Results of the present study suggest that the selection and consumption of *Bacillus*-DFM, producing a variable set of enzymes and antimicrobial compounds, may contribute to enhanced performance through improving nutrient digestibility, reducing intestinal viscosity, maintaining a beneficial gut microbiota, and promoting healthy intestinal integrity in poultry.

## Introduction

The continuous tendency to reduce the use of antibiotic growth promoters (AGP) in poultry production, due to social concern about generation of antibiotic-resistant bacteria, has resulted in the crucial necessity to find economically viable alternatives that can maintain optimal health and performance parameters under commercial conditions (1, 2). One possible substitute for AGP that has been extensively studied is the utilization of probiotics to prevent and treat gastrointestinal infections (3). The most common microorganisms used as probiotics are lactic acid bacteria (LAB) from the genus *Lactobacillus* and *Pediococcus*; however, these microorganisms required refrigeration or lyophilization to survive for long storage periods, and microencapsulation to withstand feed application, therefore adding cost to their industrial production (4). Among the microorganisms used as direct-fed microbials (DFM), *Bacillus* spores have been increasingly included as feed additives in poultry diets, due to their remarkable resistance to harsh environmental conditions, and also have a long shelf life (5, 6). Bacteria from the genus *Bacillus* are Gram-positive, rod shaped, and usual inhabitants of the soil. However, different studies have shown that *Bacillus* spores can also be present, germinate, and survive in the gastrointestinal tract (GIT) of different animal species, suggesting that these bacteria could be considered facultative anaerobes and part of the metabolically active host microbiota (7–10). Rate of survival and persistence of some *Bacillus* strains in the GIT may be related to their capacity to synthesize biofilms, thereby, protecting themselves against the harsh environmental conditions present in the gut (11). Moreover, one of the principal sources of enzymes and antibiotics from bacterial origin used by biotechnology companies are produced by different *Bacillus* strains, making this multifunctional microorganism useful inside or outside a host (12, 13).

On the other hand, the increasing consumption of poultry meat globally, along with utilization of grains such as corn for biofuel production, has led to the use of less digestible energy sources in poultry diets. Alternative cereals, such as wheat, barley, triticale, or rye, have been previously included in poultry feed (14–16). However, the incorporation of these raw materials in monogastric diets have a negative impact on growth performance due to an elevated concentration of antinutritional factors, such as the non-starch polysaccharides (NSP), in comparison to corn-based diets (17). Diets rich in NSP generate an increase in intestinal viscosity, affecting digestibility and absorption of nutrients by the intestinal surface (18). An alternative to reduce the negative effects generated by NSP is the inclusion of microbial enzymes, such as xylanase, which have been shown to reduce intestinal viscosity and *Clostridium*-associated enteritis (19). Additionally, utilization of other microbial enzymes, such as α-amylase, protease, lipase, and phytase, have demonstrated to increase degradation of low-quality proteins, improve bone quality, and enhance absorption of carbohydrates and fatty acids (20–22). In this regard, the exogenous enzymes produced by *Bacillus* spp. that may help to degrade complex antinutritional factors in poultry diets and improve nutrient absorption include cellulase (23), α-amylase (24), β-glucanase (25), α-galactosidase, β-mannanase (26), xylanase (27), protease (28), lipase (29), keratinase (30), and phytase (31). Nonetheless, it is important to mention that not all *Bacillus* bacteria synthesize the same type of enzymes, therefore require selection and characterization of adequate isolates according to the specific target substrates in the diet.

Besides the capacity of certain *Bacillus* spp. to produce enzymes and increase utilization of nutrients from different feedstuffs, spores from various *Bacillus* strains have also been included in poultry diets to control the incidence of different gastrointestinal diseases through the production of antimicrobial compounds or acting as competitive exclusion agents against *Salmonella* Typhimurium (32), *Clostridium perfringens* (33), *Escherichia coli* (34), and *Campylobacter jejuni* (35). Additionally, *Bacillus*-DFM have shown to enhance cellular and humoral immune responses by increasing the number of solitary lymphoid follicles in the intestinal mucosa, influencing the development of the gut-associated lymphoid tissue (GALT), enhancing antibody responses after vaccination, and augmenting macrophage function (36–38). Dietary supplementation with *Bacillus* spores may also have a positive effect on other beneficial bacteria populations, such as LAB, through production of subtilisin and catalase, as well as reducing pH and oxygen concentration in the gut to generate a more favorable environment (39, 40). In the case of intestinal epithelial integrity, it has been shown *in vitro* (Caco2 cells) and *ex vivo* that a *Bacillus subtilis* quorum-sensing signal molecule known as the competence and sporulation-stimulating factor (CSF), induces expression of the heat-shock protein, Hsp27, therefore enhancing protection of enterocytes against oxidative damage and preventing detrimental effects on the intestinal barrier (41). At the end, all the characteristics mentioned before support the utilization of selected *Bacillus* spp. spores as a feasible alternative to AGP, improving performance parameters through production of enzymes and maintaining an optimal health status by synthesis of antimicrobial compounds. Therefore, the purpose of the present study was to evaluate and select *Bacillus* isolates from environmental and poultry sources as candidate DFM based upon enzyme production profiles, pathogen-inhibition capacity, and biofilm synthesis, therefore, extending our understanding of the mechanism of action of *Bacillus*-DFM and its applicability in the poultry industry.

## Materials and Methods

### *Bacillus* spp. Isolation

Previous research conducted in our laboratory focused on isolation of several *Bacillus* spp. from environmental and poultry sources as described by Wolfenden et al. (42). Briefly, samples from intestinal content, fecal material, and environmental sources were collected using sterile cotton swabs and placed into sterile borosilicate tubes for transport. All samples were pasteurized by heat treatment at 70°C for 15 min to eliminate the presence of vegetative cells and allow the isolation of spore-formers only. Swabs were then plate struck on tryptic soy agar (TSA, Becton Dickinson, Sparks, MD, USA) to be able to collect individual colonies after 24 h incubation at 37°C. Additionally, all the strains used in the present study were previously selected as negative for alpha and beta hemolysis after being inoculated on TSA plates containing 50 mL/L of defibrinated sheep blood (Remel, Lenexa, KS, USA).

### *In Vitro* Determination of Enzyme Activity

Thirty-one *Bacillus* spp. isolates obtained from the Poultry Health Laboratory at the University of Arkansas were screened for production of α-amylase, protease, lipase, and phytase. All *Bacillus* strains were grown in tryptic soy broth (TSB, Becton Dickinson, Sparks, MD, USA) at 37°C for 24 h. Then the isolates were washed with a saline solution (0.9%) and centrifuged three times at 1864 × *g* for 15 min to prepare a clean inoculum. Then, 10-fold dilutions of the inoculum from each strain were plated on TSA, followed by 24 h of incubation at 37°C, to determine the cfu/mL used for assessment of enzyme activity. During the screening process, 10 μl with 10^8^ cfu/mL of each *Bacillus* strain were placed on the center of each selective media according to the enzyme under evaluation. After incubation, all plates were evaluated and the diameters of the zones of clearance were measured removing the diameter of the bacterial colony. The relative enzyme activity (REA) was determined by using the formula: REA = diameter of zone of clearance divided by the diameter of the bacterial colony in millimeters. Based on REA test organisms were categorized into excellent (REA > 5.0), good (REA > 2.0–5.0), or poor (REA < 2.0) (43). Each *Bacillus* strain was evaluated by triplicate, and values are presented in Table 1. More details about the composition of the selective media and incubation periods used to evaluate the capacity to produce each enzyme are described below.

**Table 1:** **Relative enzyme activity (REA)^a^ values produced by *Bacillus* spp. strains evaluated as enzyme producer candidates**.

*Bacillus* isolates^b^	Amylase	Protease	Lipase	Phytase
AM0902	1.0 ± 0.00	1.0 ± 0.00	1.9 ± 0.15	1.0 ± 0.00
AM0904	5.3 ± 0.19	2.7 ± 0.08	2.3 ± 0.06	1.2 ± 0.07
AM0905	5.8 ± 0.44*	3.0 ± 0.26	2.7 ± 0.17	1.6 ± 0.24
AM0908	5.3 ± 0.06	2.1 ± 0.08	2.3 ± 0.07	1.4 ± 0.10
AM0923	5.7 ± 0.19	2.8 ± 0.04	2.2 ± 0.26	1.5 ± 0.02
AM0933	5.3 ± 0.21	2.3 ± 0.09	2.1 ± 0.07	1.3 ± 0.07
AM0934	4.5 ± 0.18	3.1 ± 0.34	2.4 ± 0.35	1.2 ± 0.08
AM0938	5.0 ± 0.50	3.4 ± 0.30*	2.7 ± 0.17	2.1 ± 0.08
AM0939	3.9 ± 0.12	2.9 ± 0.44	2.2 ± 0.12	1.4 ± 0.13
AM0940	5.9 ± 0.27	1.8 ± 0.19	2.4 ± 0.21	1.4 ± 0.12
AM0941	1.0 ± 0.00	1.7 ± 0.40	2.8 ± 0.27	2.0 ± 0.12
AM1002	6.3 ± 0.12*	2.8 ± 0.15	3.0 ± 0.35*	2.1 ± 0.11
AM1010	5.7 ± 0.16	2.1 ± 0.11	2.6 ± 0.21	1.5 ± 0.12
AM1011	4.4 ± 0.30	3.0 ± 0.13	2.5 ± 0.29	1.3 ± 0.10
AM1012	6.1 ± 0.18*	2.5 ± 0.15	2.3 ± 0.17	1.4 ± 0.02
AM1013	4.1 ± 0.08	2.3 ± 0.09	2.0 ± 0.09	1.3 ± 0.05
AM1109A	2.7 ± 0.27	1.8 ± 0.10	2.2 ± 0.11	1.4 ± 0.11
AM1109B	1.8 ± 0.42	1.0 ± 0.00	2.4 ± 0.21	1.4 ± 0.07
B2/53	4.0 ± 0.64	2.7 ± 0.16	2.5 ± 0.08	1.6 ± 0.05
BL	2.2 ± 0.13	1.0 ± 0.00	1.0 ± 0.00	1.0 ± 0.00
JD17	4.0 ± 0.29	2.9 ± 0.20	2.6 ± 0.11	2.3 ± 0.15*
JD19	3.4 ± 0.33	2.1 ± 0.17	2.2 ± 0.12	1.5 ± 0.01
NP001	4.3 ± 0.19	2.3 ± 0.14	1.9 ± 0.11	1.1 ± 0.04
NP002	3.0 ± 0.40	2.3 ± 0.29	2.1 ± 0.11	1.2 ± 0.12
NP117B	2.7 ± 0.48	3.0 ± 0.06	2.1 ± 0.14	1.3 ± 0.12
NP121	3.1 ± 0.46	2.2 ± 0.13	2.0 ± 0.09	1.5 ± 0.14
NP122	4.7 ± 0.36	2.8 ± 0.40	2.3 ± 0.15	1.3 ± 0.12
NP124	1.6 ± 0.40	2.1 ± 0.29	2.2 ± 0.12	1.1 ± 0.00
NP126	3.3 ± 0.23	2.5 ± 0.15	2.2 ± 0.12	1.2 ± 0.07
MM65	3.8 ± 0.31	1.0 ± 0.00	3.0 ± 0.22	2.5 ± 0.06*
RW41	4.2 ± 0.88	1.3 ± 0.11	2.0 ± 0.04	1.2 ± 0.04

*Identified bacterial strains as superior enzyme producers with a higher REA value, P < 0.05.

*^a^REA was calculated dividing the diameter of area of clearance by the diameter of the *Bacillus* colony. Organism were classified as excellent (REA > 0.5), good (REA > 2.0–5.0), or poor (REA < 2.0) enzyme producers. Data expressed as mean ± SE*.

*^b^All Bacillus spp. isolates were tested by triplicate*.

### Production of Amylase

To determine α-amylase enzyme activity, a starch agar media was used and consisted of 10 g of tryptone, 3 g of soluble starch, 5 g of KH_2_PO_4_, 10 g of yeast extract, 15 g of noble agar, and 1000 mL of distilled water. The starch media was autoclaved at 121°C for 15 min and poured in Petri dishes when the temperature reaches 50°C. Then each tested *Bacillus* strain was inoculated and incubated at 37°C for 48 h. For visualization of the zone of clearance, all Petri dishes were flooded with 5 mL of Gram’s iodine solution (24).

### Production of Protease

For evaluation of protease activity, a skim milk agar media was prepared containing 25 g of skim milk, 25 g of noble agar, and 1000 mL of distilled water. The mixture was stirred thoroughly and autoclaved at 121°C for 15 min. For plating, the skim milk agar solution was held in a water bath at 50°C, and then it was poured quickly into plates. Each *Bacillus* strain was inoculated on Petri dishes and incubated at 37°C for 24 h to observe if a zone of clearance was developed (44).

### Production of Lipase

Lipase activity was assessed using the Spirit blue agar media (Difco Laboratories, Detroit, MI, USA) composed by 10 g of pancreatic digest of casein, 5 g of yeast extract, 20 g of noble agar, and 0.15 g of the die spirit blue. A total of 35 g spirit blue agar were used per 1000 mL of distilled water. The media was sterilized at 121°C for 15 min and cooled to 50°C in a water bath, before being mixed with 30 mL of a lipoidal solution prepared with 100 mL of olive oil, 1 mL of polysorbate 80, and 400 mL of warm water (60°C). Plates were inoculated and incubated at 37°C for 24 h, before the determination of a zone of clearance around each bacterial colony (45).

### Production of Phytase

For determination of phytase activity *Bacillus* isolates were screened in a medium that contained: 10 g dextrose, 0.3 g (NH_4_)_2_SO_4_, 0.5 g MgSO_4_, 0.1 g CaCl_2_, 0.01 g MnSO_4_, 0.01 g FeSO_4_, 5 g Na-phytate, and 20 g of noble agar per 1000 mL of distilled water. The phytate media was autoclaved at 121°C for 15 min and poured into Petri dishes when the temperature reached 50°C. Isolates were inoculated and incubated at 37°C for a maximum of 120 h to evaluate if a zone of clearance was generated surrounding the tested bacterial strains (46, 47).

### *In Vitro* Assessment of Antimicrobial Activity against *Salmonella enterica* serovar Enteritidis and *Escherichia coli*

Thirty-one *Bacillus* spp. strains were screened by triplicate for *in vitro* antimicrobial activity against *Salmonella enterica* serovar Enteritidis (*S*. Enteritidis), bacteriophage type 13A, obtained from the USDA National Veterinary Services Laboratory (Ames, IA, USA), and a wild-type poultry field strain *E. coli*, as reported previously by Wolfenden et al. (42). Briefly, 10 μl with 10^8^ cfu/mL of each *Bacillus* isolate were placed on the center of TSA plates and incubated for 24 h at 37°C. Then, the Petri dishes with visible *Bacillus* colonies were overlaid with a TSA soft agar containing either 10^6^ cfu/mL of *S*. Enteritidis or *E. coli*. After aerobic incubation for 24 h at 37°C, all plates were observed and the diameters of the zones of inhibition were measured removing the diameter of the bacterial colony.

### *In Vitro* Assessment of Antimicrobial Activity against *Clostridioides difficile*

All tested *Bacillus* spp. isolates were cultured aerobically overnight on TSA plates and screened for *in vitro* antimicrobial activity against *Clostridioides difficile* (*C. difficile*) ATCC 9689D, formerly known as *Clostridium difficile* (48). Briefly, 10 μl with 10^8^ cfu/mL of each *Bacillus* strain were placed in the center of TSA plates. After 24 h of incubation at 37°C, the plated samples were overlaid with TSA containing sodium thioglycolate (0.25 g/L) and 10^6^ cfu/mL of *C. difficile*. Then, all plates were incubated anaerobically using a BD GasPak EZ container system (Becton Dickinson, Sparks, MD, USA). After 24 h of incubation at 37°C, plates were evaluated for the presence of zones of inhibition, and the diameter of the inhibition zone was measured as mentioned above for *S*. Enteritidis and *E. coli* antimicrobial activity evaluation.

### Biofilm Assay

To determine biofilm synthesis a previously published crystal violet staining method was used with slight modifications (49). Briefly, *Bacillus* isolates were grown in TSB overnight at 37°C, and 10 μl of each strain were inoculated in 0.5 mL of Casein-Mannitol (CM) broth in 1.5 mL polypropylene tubes. The CM broth contained per liter: 10 g casein digest (Sigma-Aldrich Co., St. Louis, MO, USA) and 10 g d-mannitol. After 12 h of incubation of the CM broth at 37°C without shaking, the liquid supernatant was removed and the tubes were gently rinsed with distilled water. Then, 1 mL of a 1% w/v crystal violet solution was added to the tubes to stain the cells adhered to the walls forming a ring. After 25 min, the crystal violet solution was removed, and the tubes were washed with distilled water. The qualitative measurement of biofilm synthesis was based on color intensity and size of the adherent crystal violet ring with a score ranging from negative (−) to strong (++) biofilm formation described by Fall et al. (50). Additionally, all samples were scored by the same person to minimize variability and maintain results consistency.

### Identification of *Bacillus*-DFM Candidates

*Bacillus* spp. strains laboratory identified as AM1002, AM0938, and JD17 were selected as superior enzyme producers based on their enzyme production profile. These candidates were identified and characterized based on biochemical evaluation tests using a bioMerieux API 50 CHB test kit (bioMerieux, Marcy l’Etoile, FRA). Selected candidates were also subjected to 16S rRNA sequence analysis in a specialized laboratory using Sherlock^®^ DNA microbial analysis software and database (Midi labs, Newark, DE, USA). Briefly, the 16S rRNA gene was PCR amplified from genomic DNA isolated from pure bacterial colonies. Primers used are universal 16S primers that correspond to positions 0005F and 0531R for a 500 bp sequence and 0005F and 1513R for the 1500 bp sequence. Amplification products were purified from excess primers and dNTPs and checked for quality and quantity by running a portion of the products on an agarose gel. Cycle sequencing of the 16S rRNA amplification products was carried out using DNA polymerase and dye terminator chemistry. Excess dye-labeled terminators were then removed from the sequencing reactions. The samples were electrophoresed on either a 3130 or 3130xl Genetic Analyzer.

### Statistical Analysis

Data from all measurements were subjected to one-way analysis of variance as a completely randomized design using the General Linear Models procedure of SAS (version 9.1, SAS Institute Inc., Cary, NC, USA) (51). Means were separated with Duncan’s multiple-range test and considered significant at *P* < 0.05. Data were reported as mean ± SE.

## Results

### Determination of *In Vitro* Enzyme Activity

**Figure 1:**
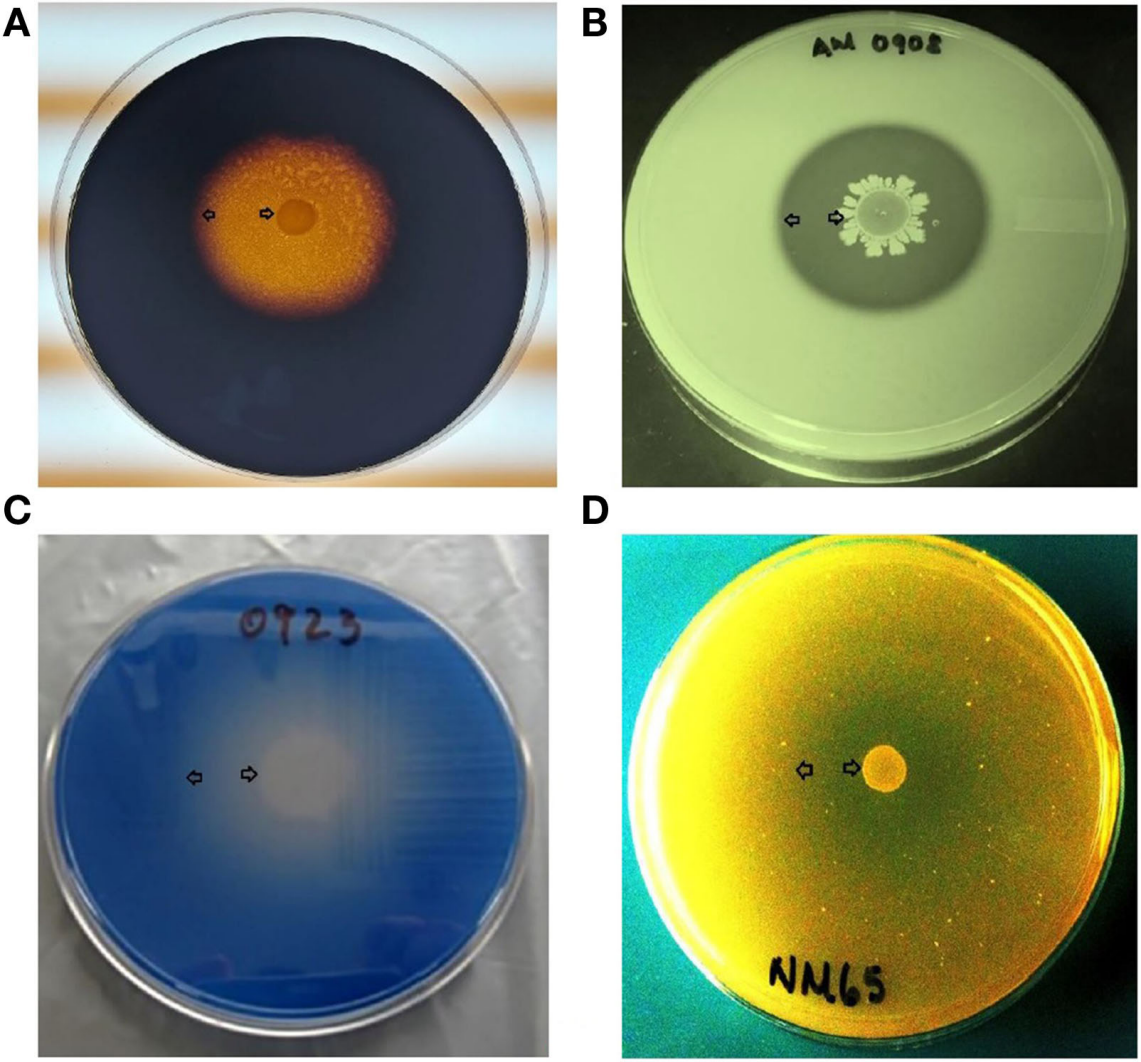
**Representative examples of microbial enzyme activity using a different selective media for each enzyme under evaluation**. An area of clearance around a bacterial colony can be observed, representing enzyme production of **(A)** amylase, **(B)** protease, **(C)** lipase, and **(D)** phytase. All *Bacillus* spp. strains were screened by triplicate. Arrows indicate the bacterial colony and the outer limit of the zone of clearance.

*Bacillus* spores were isolated by heat treatment of intestinal, fecal, and environmental samples, eliminating the presence of vegetative cells. Although enzyme activity was detected for the majority of the strains, there were considerable differences in their REA values. Three of the 31 screened *Bacillus* spp. strains showed a significantly higher REA value for amylase production in comparison to other bacterial colonies. Isolates AM1002, AM1012, and AM0905 obtained REA values of 6.3, 6.1, and 5.8, respectively, all of them categorizing these *Bacillus* isolates as excellent amylase producers (REA > 5.0). In the case of protease activity, strain AM0938 showed a REA value of 3.4 which is considered good (REA > 2.0–5.0), surpassing the enzyme activity values of all other screened strains. Lipase synthesis was significantly superior in the isolate AM1002 (REA = 3.0), meanwhile, phytase production was classified as good for the strains JD17 (REA = 2.3) and MM65 (REA = 2.5) in comparison to the other screened *Bacillus* spp. isolates. A complete description of the enzyme activity profile of all the evaluated isolates and the appearance of each selective media are presented in Table 1 and Figure 1, respectively.

### *In Vitro* Evaluation of Antimicrobial Activity

**Figure 2:**
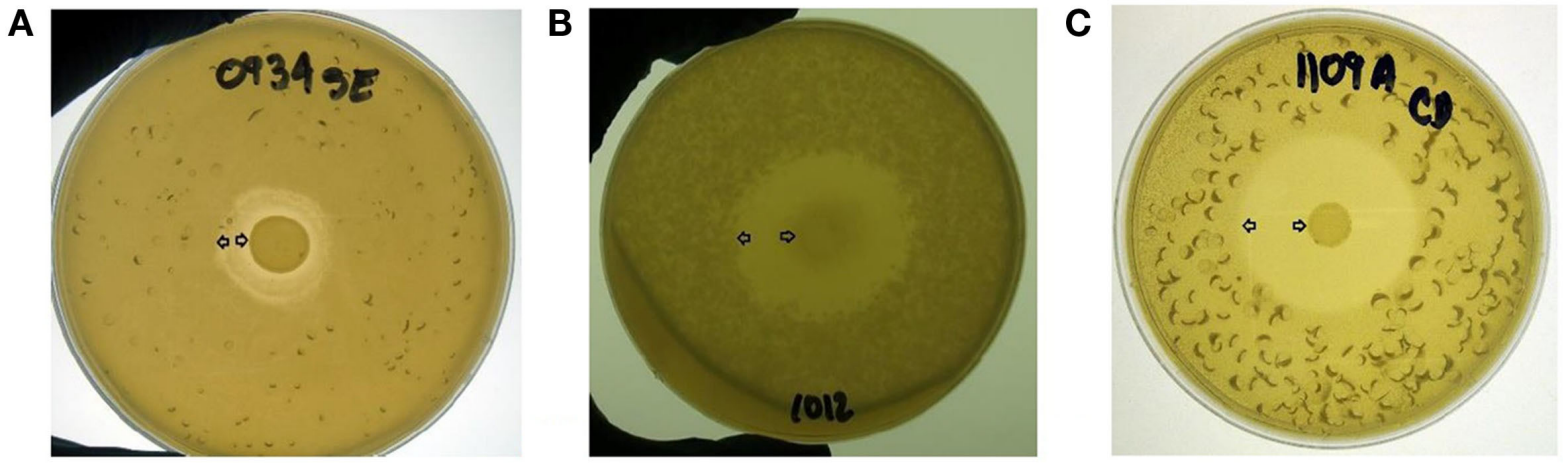
**Evaluation of antimicrobial activity from different *Bacillus* spp. isolates using an overlay method**. A zone of inhibition is shown surrounding a tested bacterial colony located in the middle of the plate against **(A)** *S*. Enteritidis, **(B)** *E. coli*, and **(C)** *C. difficile*. All *Bacillus* spp. strains were screened by triplicate. Arrows indicate the bacterial colony and the outer limit of the zone of inhibition.

An overlay method was used to assess the production of antimicrobial compounds by the 31 *Bacillus* strains against Gram-positive and Gram-negative enteropathogens (Table 2; Figure 2). Although antimicrobial activity was observed in a greater number of isolates, individual differences were evident in the degree of inhibition and spectrum of activity. In the case of *S*. Enteritidis, isolate NP122 generated the largest diameter of the zone of inhibition with 13.7 mm, followed by the strain AM0904 with a inhibition diameter of 12.0 mm. Activity against *E. coli* was more evident in isolates AM1010 and AM1012, both with a diameter of clearance of 20 mm. Interestingly, *C. difficile* was the most susceptible microorganism in the presence of almost all *Bacillus* spp. strains, with an average zone of inhibition of 19 mm for the 31 isolates, where the strain AM1010 produced larger pathogen-inhibition activity with a diameter of clearance of 28 mm.

**Table 2:** **Evaluation of antimicrobial activity^a^ and biofilm synthesis^b^ of different *Bacillus* spp. isolates**.

*Bacillus* isolates	*S*. Enteritidis (mm)	*E. coli* (mm)	*C. difficile* (mm)	Biofilm formation
AM0902	0.0 ± 0.00	0.0 ± 0.00	0.0 ± 0.00	+
AM0904	12.0 ± 0.38*	16.0 ± 2.31	26.0 ± 1.86	+
AM0905	6.7 ± 0.67	14.0 ± 1.15	20.3 ± 1.67	++
AM0908	6.0 ± 0.56	4.3 ± 0.33	22.0 ± 2.31	+
AM0923	7.7 ± 0.30	10.0 ± 3.06	24.0 ± 3.06	+
AM0933	1.3 ± 0.33	4.0 ± 0.58	10.0 ± 1.15	++
AM0934	6.3 ± 0.40	8.7 ± 1.76	22.7 ± 2.40	+
AM0938	8.0 ± 1.15	10.0 ± 2.00	22.0 ± 2.00	+
AM0939	6.3 ± 0.88	8.3 ± 1.33	26.0 ± 2.60	+
AM0940	8.0 ± 1.12	10.3 ± 1.67	21.0 ± 1.76	++
AM0941	0.7 ± 0.27	0.0 ± 0.00	0.0 ± 0.00	++
AM1002	5.7 ± 0.58	8.7 ± 1.76	16.0 ± 2.08	++
AM1010	8.0 ± 1.10	20.0 ± 1.45*	28.0 ± 2.67*	+
AM1011	8.5 ± 0.90	10.7 ± 1.76	20.3 ± 2.33	++
AM1012	8.7 ± 0.88	20.0 ± 2.19*	10.0 ± 1.75	++
AM1013	4.0 ± 1.15	10.0 ± 1.15	22.0 ± 1.15	+
AM1109A	10.3 ± 1.20	12.0 ± 1.50	24.0 ± 1.11	++
AM1109B	0.3 ± 0.33	0.0 ± 0.00	14.7 ± 1.62	++
B2/53	10.3 ± 1.20	12.0 ± 0.58	26.0 ± 3.08	+
BL	0.0 ± 0.00	4.0 ± 0.52	10.0 ± 2.00	+
JD17	6.3 ± 0.33	10.0 ± 1.15	20.6 ± 3.53	+
JD19	2.0 ± 0.58	2.7 ± 0.67	19.0 ± 1.72	+
NP001	8.0 ± 0.88	6.0 ± 0.58	12.0 ± 1.13	+
NP002	4.3 ± 1.33	6.0 ± 1.10	20.7 ± 2.40	+
NP117B	2.7 ± 0.67	6.0 ± 1.15	18.0 ± 3.46	+
NP121	2.3 ± 0.33	14.0 ± 3.06	16.0 ± 2.31	+
NP122	13.7 ± 1.86*	12.0 ± 2.00	26.0 ± 4.16	++
NP124	6.0 ± 1.73	12.0 ± 1.86	22.0 ± 2.03	+
NP126	0.3 ± 0.30	2.0 ± 1.89	21.7 ± 1.76	+
MM65	8.0 ± 0.55	10.0 ± 1.15	20.3 ± 1.45	++
RW41	5.7 ± 0.88	10.0 ± 2.00	22.0 ± 2.28	+

*Identified bacterial strains with the enhanced antimicrobial activity, P < 0.05.

*^a^Represents the diameter of the zone of inhibition observed at 24 h of incubation without the diameter of the bacterial colony. Data expressed as mean ± SE*.

*^b^The qualitative measurement of biofilm synthesis was based on color intensity and size of the adherent crystal violet ring with a score ranging from negative (−) to strong (++) biofilm formation. All *Bacillus* spp. isolates were tested by triplicate*.

### Biofilm Synthesis

**Figure 3:**
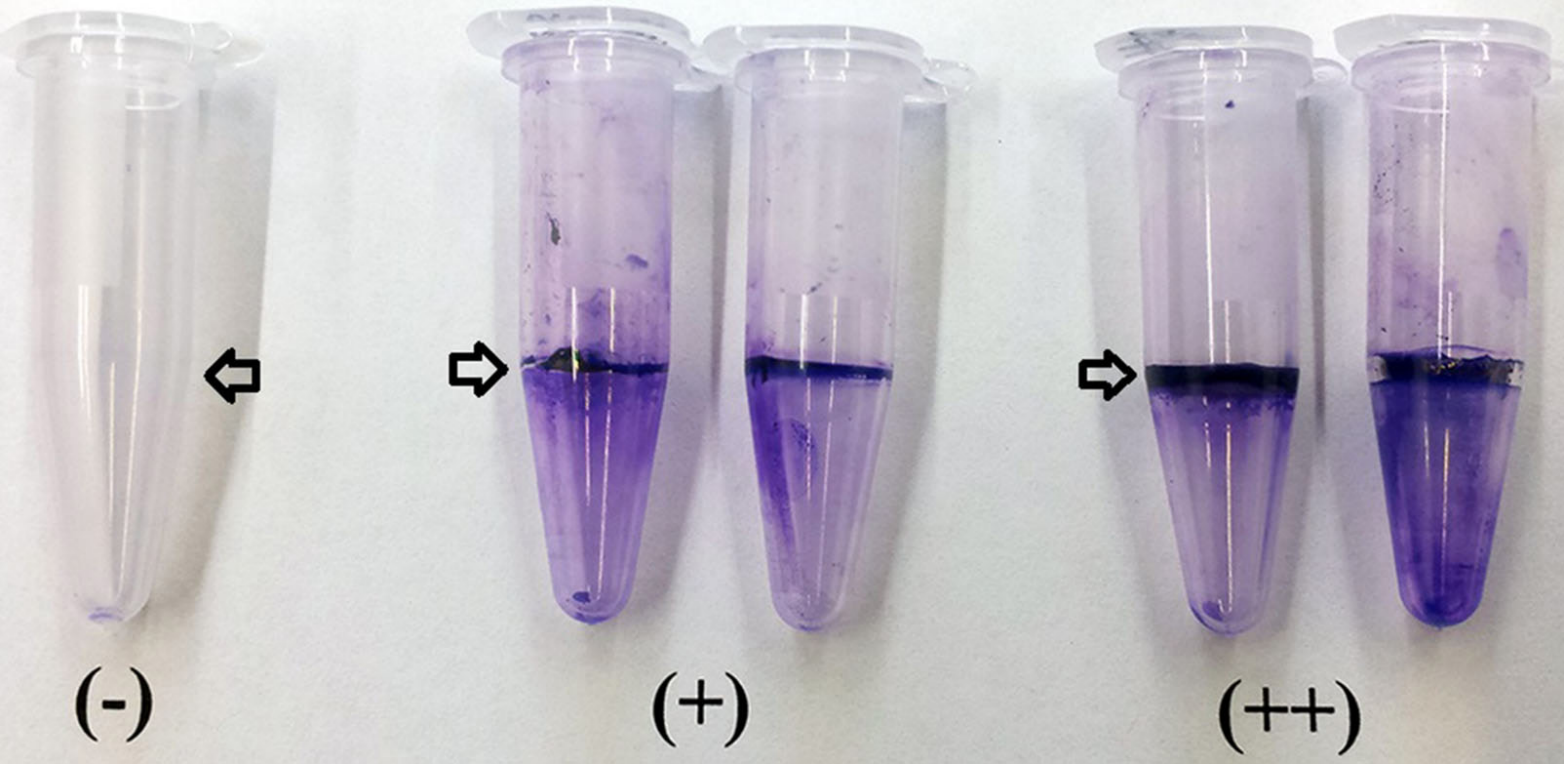
**Determination of biofilm synthesis was performed using a crystal violet staining method**. Measurement of biofilm synthesis was based on color intensity and size of the adherent crystal violet ring with a score ranging from negative (−) to strong (++) biofilm formation. All *Bacillus* spp. strains were screened by triplicate. Arrows indicate the presence or absence of the biofilm ring.

Biofilm production was evaluated by generation of an adherent crystal violet-stained ring in polypropylene tubes. All the screened *Bacillus* spp. strains produced biofilms; however, isolates AM0905, AM0933, AM0940, AM0941, AM1002, AM1011, AM1012, AM1109A, AM1109B, NP122, and MM65 were identified as strong biofilm formers with a wider and more colorful intense ring of adherence present on the wall of the test tubes (Table 2; Figure 3).

### Characterization and Selection of *Bacillus*-DFM Candidates

Based on the REA results, three *Bacillus-*DFM candidates were selected with excellent to good REA values for each of the evaluated enzymes. These candidates were then identified and characterized using a bioMerieux API 50 CHB test kit (bioMerieux, Marcy l’Etoile, France). This set of biochemical tests classified *Bacillus* spp. strains based on their capacity to metabolize 49 different carbohydrates (Table 3). According to the fermentation profile, all isolates were categorized as *B. subtilis/Bacillus amyloliquefaciens* with an identification percentage of 99.0% or higher. To further assist in identification of the strains, each isolate was also subjected to 16S rRNA sequence analysis in a specialized laboratory (Midi labs, Newark, DE, USA). 16S rRNA sequence analysis identified isolate AM1002, as *B. subtilis* (GenBank Match: 100%, accession number AB201120); AM0938 as *B. amyloliquefaciens* (GenBank Match: 100%, accession number GU191912); and JD17 as *B. amyloliquefaciens* (GenBank Match: 100%, accession number GU191912). These three isolates have been deposited at the Agricultural Research Service Culture Collection (NRRL Peoria, IL, USA) by Pacific Vet Group USA, Inc., with the NRRL numbers: AM1002/B-67143; AM0938/B-67144; and JD17/B-67142.

**Table 3:** **Characterization and identification of selected *Bacillus*-DFM candidate strains based on biochemical carbohydrate metabolism tests**.^a^^,^^b^

Item	AM1002	AM0938	JD17
Amidon (starch)	+	+	+
Amygdalin	+	+	+
Arbutin	+	+	+
d-Adonitol	−	−	−
d-Arabinose	−	−	−
d-Arabitol	−	−	−
d-Cellobiose	+	+	+
d-Fructose	+	+	+
d-Fucose	−	−	−
d-Galactose	−	−	−
d-Glucose	+	+	+
d-Lactose (bovine origin)	+	+	+
d-Lyxose	−	−	−
d-Maltose	+	+	+
d-Mannitol	+	+	+
d-Mannose	+	+	+
d-Melezitose	−	−	−
d-Melibiose	+	−	+
d-Raffinose	+	+	+
d-Ribose	+	+	+
d-Saccharose (sucrose)	+	+	+
d-Sorbitol	+	+	−
d-Tagatose	−	−	−
d-Trehalose	+	+	+
d-Turanose	−	−	−
Dulcitol	−	−	−
d-Xylose	+	+	+
Erythritol	−	−	−
Esculin (ferric citrate)	+	+	+
Gentibiose	+	+	−
Glycerol	+	+	+
Glycogen	+	+	+
Inositol	+	+	+
Inulin	+	−	−
l-Arabinose	+	+	+
l-Arabitol	−	−	−
l-Fucose	−	−	−
l-Rhamnose	−	−	−
l-Sorbose	−	−	−
l-Xylose	−	−	−
Methyl-αd-glucopyranoside	+	+	+
Methyl-αd-mannopyranoside	−	−	−
Methyl-βd-xylopyranoside	−	−	−
*N*-Acetylglucosamine	−	−	−
Potassium 2-ketogluconate	−	−	−
Potassium 5-ketogluconate	−	−	−
Potassium gluconate	−	−	−
Salicin	+	+	+
Xylitol	−	−	−

*^a^BioMerieux API50 CHB test kit (bioMerieux, Marcy l’Etoile, France)*.

*^b^Different scores (+ or −) reflect the capacity of the tested *Bacillus* spp. isolate to ferment an specific carbohydrate or carbohydrate derivative*.

## Discussion

Nowadays, poultry diets include a variety of ingredients from different plant and animal sources. Due to an increasing demand of cereal grains for production of biofuels, rising corn prices have had a direct impact on diet costs (52). Consequently, the necessity to reduce costs of production has required the inclusion of less digestible and more available raw materials in poultry diets. Distillers’ dried grains with solubles (DDGS) are usually available to be included in the ingredient matrix, as a result of the continuous development of the ethanol industry (53). However, the main concern with the inclusion of high percentages of DDGS in poultry diets is related to its variable nutritional content and nutrient digestibility. Moreover, it has been observed that high levels of DDGS in the diet could act as a predisposing factor for presentation of necrotic enteritis (54). On the other hand, alternative grains, such as wheat, barley, rye, and sorghum, conform a different group of unconventional feed ingredients that have increased their participation in poultry diets as energy sources; nevertheless, it is important to mention that these feedstuffs often contain a higher concentration of antinutritional factors, such as NSP, in comparison to corn (55). An elevated concentration of arabinoxylans or β-glucans in the intestinal content has been related to reduced nutrient absorption and increased intestinal viscosity and microbial growth (56). Therefore, as an alternative to improve nutrient utilization and increase flexibility of the ingredient matrix used in poultry diets, multiple researchers have been evaluating the inclusion of different exogenous feed enzymes either alone or in diverse combinations (57). It has been well established that incorporation of carbohydrases (xylanase, β-glucanase, or amylase) and phytase can reduce the adverse impact of antinutritional factors in monogastric animals fed with different raw materials (58). Additionally, a growing interest on the reduction of environmental pollution generated by livestock production has been one of the principal targets supporting the inclusion of enzymes in animal feed (59). Nevertheless, research results have been variable due to the different sources of exogenous enzymes under evaluation. Some of these enzymes are denatured at acidic pH (proventriculus) or do not resist high temperatures commonly used during feed pelletization. One of the principal sources of microbial enzymes is produced by bacteria from the genus *Bacillus* (24, 27). For this reason in the present study, 31 *Bacillus* spp. were screened for production of amylase, protease, lipase, and phytase (Table 1). Three strains were selected based on superior REA values on at least one of the enzymes under evaluation. These results demonstrate that not all *Bacillus* spp. synthesize the same type of enzymes over time, suggesting that this capacity is a strain-specific characteristic (Figure 1). The combination and feed inclusion of these superior enzyme producer isolates as a *Bacillus*-DFM cocktail has been previously evaluated during *in vivo* experiments with broiler chickens and turkeys (9, 60). In these experiments, results showed that consumption of the DFM significantly improved performance parameters, intestinal viscosity, bacterial translocation, and bone quality in poultry fed with a rye-based diet containing high amounts of NSP.

On the other hand, despite of the success showed by the development of the LAB probiotics for use in commercial poultry, there is still an urgent necessity for commercial DFM that are shelf-stable, cost-effective, and feed-applicable to increase widespread utilization of viable substitutes of AGP in the poultry industry. In this regard, *Bacillus* spp. spores have been isolated from the GIT of multiple animal species, including poultry and pigs suggesting that this microorganism could be an active member of the host microbiota (11, 61). Moreover, some *Bacillus* spp. endospores have been extensively studied as DFM, showing to be a safe and reliable prophylactic tool to diminish the presentation of gastrointestinal diseases in livestock and humans (62–64). In the present study, the majority of the tested *Bacillus* spp. strains showed antimicrobial activity against different food-borne pathogens, including *S*. Enteritidis (25/31) and *E. coli* (27/31). This could be the result of the capacity of some *Bacillus* to synthesize antimicrobial compounds, compete for nutrients, and/or change the environmental conditions of the media (Figure 2). Furthermore, it was remarkable to observe that the most susceptible enteropathogen to the presence of almost all *Bacillus* isolates was *C. difficile* (28/31). This anaerobic sporeformer bacteria is the principal etiological agent of nosocomial diarrhea in patients under antibiotic therapy, and it has also been isolated from animals and retail meat (65, 66). Therefore, these results suggest that utilization of selected *Bacillus*-DFM may be a suitable alternative to reduce the incidence of bacterial gastrointestinal diseases in humans and animals, including cases of *C. difficile* infection. However, as observed in the enzyme-production profile, the ability to produce antimicrobial compounds appears to be a specific feature for each *Bacillus* spp. isolate (Table 2).

In the case of biofilm formation, it is possible that this polysaccharide structure served as a mechanism of survival for some *Bacillus* isolates to resist the harsh environmental conditions of the GIT. Additionally, generation of biofilms could help *Bacillus* cells to be attached to the gut epithelia, therefore, increasing their persistence in the intestinal mucosa, as well as, preventing adherence of enteropathogens as suggested by Barbosa et al. (11). Results of the biofilm assay showed that 11 of 31 *Bacillus* spp. synthesized a thicker and stronger adherent layer, therefore classifying this isolates as superior biofilm formers. Previous studies from our laboratories has evaluated germination, distribution, and persistence of *B. subtilis* spores in the GIT of poultry, and it was observed that spores from the isolate NP122 which synthesized biofilms, persisted for 120 h after a single gavage dose, that is longer than the estimated half-life, based on gut-passage time of the digesta in poultry (10). This finding could be an important strain-specific characteristic influencing the viability of different *Bacillus* candidates in the GIT; however, more studies need to be conducted to confirm this hypothesis.

In summary, our results confirm that *Bacillus* spp. isolates differ in their capacity to produce enzymes, antimicrobial compounds, and biofilms even if they are from the same species. Therefore, an exhaustive selection process must be performed according to the purpose the DFM is going to be used for. *Bacillus* strains selected as superior enzyme producers were different from the isolates showing the highest antimicrobial activity; however, all *Bacillus* isolates showed certain pathogen-inhibition activity. As observed in previous *in vivo* experiments in poultry consuming rye-based diets, it is expected that the consumption of the *Bacillus*-DFM candidate selected in this study, based on enzyme activity, may contribute to enhanced performance parameters by improving nutrient digestibility, maintaining a balanced microbiota, and promoting healthy intestinal integrity in poultry consuming conventional corn-based diets and/or diets containing alternative feed ingredients with a higher content of NSP.

## Author Contributions

JL: conception and design, acquisition of data, and drafting of manuscript. XH-V: drafting the article or revising it critically for important intellectual content. RW: acquisition of data and drafting the article. JV: acquisition of data. AW: acquisition of data. AM: acquisition of data. LB: acquisition of data. BH: approval of the version to be submitted and any revised version. GT: conception and design, acquisition of data, analysis and interpretation of data, and drafting of manuscript.

## Conflict of Interest Statement

The authors declare that the research was conducted in the absence of any commercial or financial relationships that could be construed as a potential conflict of interest.

## References

[1] Alvarez-Olmos MI Oberhelman RA. Probiotic agents and infectious diseases: a modern perspective on a traditional therapy. Clin Infect Dis (2001) 32:1567–76. 10.1086/320518 11340528

[2] Boyle EC Bishop JL Grassl GA Finlay BB *Salmonella*: from pathogenesis to therapeutics. J Bacteriol (2007) 189:1489–95. 10.1128/JB.01730-06 17189373 PMC1855715

[3] Higgins S Wolfenden A Tellez G Hargis B Porter T. Transcriptional profiling of cecal gene expression in probiotic-and *Salmonella*-challenged neonatal chicks. Poult Sci (2011) 90:901–13. 10.3382/ps.2010-00907 21406379

[4] Tellez G Pixley C Wolfenden R Layton S Hargis B Probiotics/direct fed microbials for *Salmonella* control in poultry. Food Res Int (2012) 45:628–33. 10.1016/j.foodres.2011.03.047

[5] Cartman ST La Ragione RM Woodward MJ Bacterial spore formers as probiotics for poultry. Food Sci Technol Bull (2007) 4:21–30. 10.1616/1476-2137.14897

[6] Vreeland RH Rosenzweig WD Powers DW. Isolation of a 250 million-year-old halotolerant bacterium from a primary salt crystal. Nature (2000) 407:897–900. 10.1038/35038060 11057666

[7] Hoa TT Duc LH Isticato R Baccigalupi L Ricca E Van PH Fate and dissemination of *Bacillus subtilis* spore in a murine model. Appl Environ Microbiol (2001) 67:3819–23. 10.1128/AEM.67.9.3819-3823.2001 11525972 PMC93096

[8] Hong HA Khaneja R Tam NM Cazzato A Tan S Urdaci M *Bacillus subtilis* isolated from the human gastrointestinal tract. Res Microbiol (2009) 160:134–43. 10.1016/j.resmic.2008.11.002 19068230

[9] Latorre JD Hernandez-Velasco X Kogut MH Vicente JL Wolfenden R Wolfenden A Role of a *Bacillus subtilis* direct-fed microbial on digesta viscosity, bacterial translocation, and bone mineralization in turkey poults fed with a rye-baed diet. Front Vet Sci (2014) 1:26 10.3389/fvets.2014.00026 26664925 PMC4668850

[10] Latorre JD Hernandez-Velasco X Kallapura G Menconi A Pumford NR Morgan MJ Evaluation of germination, distribution, and persistence of *Bacillus subtilis* spore through the gastrointestinal tract of chickens. Poult Sci (2014) 93:1793–800. 10.3382/ps.2013-03809 24812242

[11] Barbosa TM Serra CR La Ragione RM Woodward MJ Henriques AO. Screening for *Bacillus* isolates in the broiler gastrointestinal tract. Appl Environ Microbiol (2005) 71:968–78. 10.1128/AEM.71.2.968-978.2005 15691955 PMC546680

[12] Priest FG Extracellular enzyme synthesis in the genus *Bacillus*. Bacteriol Rev (1977) 41:711–53. 334155 10.1128/br.41.3.711-753.1977 PMC414021

[13] Azevedo EC Rios EM Fukushima K Campos-Takaki GM Bacitracin production by a new strain of *Bacillus subtilis*. Appl Biochem Biotech (1993) 42:1–7. 10.1007/BF02788897 8215347

[14] Leeson S Proulx J Enzymes and barley metabolizable energy. J Appl Poult Res (1994) 3:66–8. 10.1093/japr/3.1.66

[15] Bedford MR Schulze H. Exogenous enzymes for pigs and poultry. Nutr Res Rev (1998) 11:91–114. 10.1079/NRR19980007 19087461

[16] Mendes A Ribeiro T Correia B Bule P Macas B Falcao L Low doses of exogenous xylanase improve the nutritive value of triticale-based diets for broilers. J Appl Poult Res (2013) 22:92–9. 10.3382/japr.2012-00610

[17] Choct M Hughes RJ Wang J Bedford MR Morgan AJ Annison G. Increased small intestinal fermentation is partly responsible for the anti-nutritive activity of non-starch polysaccharides in chickens. Br Poult Sci (1996) 37:609–21. 10.1080/00071669608417891 8842468

[18] Annison G The role of wheat non-starch polysaccharides in broiler nutrition. Aust J Agric Res (1993) 44:405–22. 10.1071/AR9930405c

[19] Guo S Liu D Zhao X Li C Guo Y. Xylanase supplementation of a wheat-based diet improved nutrient digestion and mRNA expression of intestinal nutrient transporters in broiler chickens infected with *Clostridium perfringens*. Poult Sci (2014) 93:94–103. 10.3382/ps.2013-03188 24570428

[20] Meng X Slominski B Guenter W. The effect of fat type, carbohydrase, and lipase addition on growth performance and nutrient utilization of young broilers fed wheat-based diets. Poult Sci (2004) 83:1718–27. 10.1093/ps/83.10.1718 15510559

[21] Woyengo TA Nyachoti CM Review: supplementation of phytase and carbohydrates to diets for poultry. Can J Anim Sci (2011) 91:177–92. 10.4141/CJAS2012-017

[22] Murugesan GR Romero LF Persia ME Effects of protease, phytase and a *Bacillus sp*. direct-fed microbial on nutrient and energy digestibility, ileal brush border digestive enzyme activity and cecal short-chain fatty acid concentration in broiler chickens. PLoS One (2014) 9(7):e101888 10.1371/journal.pone.0101888 25013936 PMC4094469

[23] Hendricks CW Doyle JD Hugley B. A new solid medium for enumerating cellulose-utilizing bacteria in soil. Appl Environ Microbiol (1995) 61:2016–9. 16535032 10.1128/aem.61.5.2016-2019.1995 PMC1388450

[24] Ibrahim SE El-Amin HB Hassan EN Sulieman AME Amylase production on solid state fermentation by *Bacillus* spp. Food Public Health (2012) 2:30–5. 10.5923/j.fph.20120201.06

[25] Aono R Sato M Yamamoto M Horikoshi K Isolation and partial characterization of an 87-kilodalton β-1,3 glucanase from *Bacillus circulans* IAM1165. Appl Environ Microbiol (1992) 58:520–4. 1610176 10.1128/aem.58.2.520-524.1992 PMC195278

[26] Talbot G Sygusch J Purification and characterization of thermostable β-mannanase and α-galactosidase from *Bacillus stearothermophilus*. Appl Environ Microbiol (1990) 56:3505–10. 2176449 10.1128/aem.56.11.3505-3510.1990 PMC185002

[27] Monisha R Uma MV Krishna Murthy V Partial purification and characterization of *Bacillus pumilus* xylanase from soil source. KATSU (2009) 5:137–48.

[28] Olajuyigbe FM Ajele JO Production dynamics of extracellular protease from *Bacillus* species. Afr J Biotechnol (2005) 4:776–9.

[29] Shah KR Bhatt SA Purification and characterization of lipase from *Bacillus subtilis* Pa2. J Biochem Tech (2011) 3:292–5.

[30] Mazotto AM Coelho RR Lage-Cedrola SM Lima MF Couri S Paraguai de Souza E Keratinase production by three *Bacillus* spp. using feather meal and whole feathers as substrate in a submerged fermentation. Enzyme Res (2011) 2011:523780. 10.4061/2011/523780 21822479 PMC3148598

[31] Choi YM Suh HJ Kim JM Purification and properties of extracellular phytase from *Bacillus* spp. KHU-10. J Protein Chem (2001) 20:287–92. 10.1023/A:1010945416862 11594462

[32] Shivaramaiah S Pumford N Morgan M Wolfenden R Wolfenden A Torres-Rodriguez A Evaluation of *Bacillus* species as potential candidates for direct-fed microbials in commercial poultry. Poult Sci (2011) 90:1574–80. 10.3382/ps.2010-00745 21673174

[33] Tactacan GB Schmidt JK Miille MJ Jimenez DR A *Bacillus subtilis* (QST 713) spore-based probiotic for necrotic enteritis control in broiler chickens. J Appl Poult Res (2013) 22:825–31. 10.3382/japr.2013-00730

[34] La Ragione RM Casula G Cutting SM Woodward MJ. *Bacillus subtilis* spores competitively exclude *Escherichia coli* O78:K80 in poultry. Vet Microbiol (2001) 79:133–42. 10.1016/S0378-1135(00)00350-3 11230935

[35] Svetoch EA Stern NJ Eruslanov BV Kovalev YN Volodina LI Perelygin VV Isolation of *Bacillus circulans* and *Paenibacillus polymyxa* strains inhibitory to *Campylobacter jejuni* and characterization of associated bacteriocins. J Food Prot (2005) 68:11–7. 15690798 10.4315/0362-028x-68.1.11

[36] Rhee KJ Sethupathi P Driks A Lanning DJ Knight KL Role of commensal bacteria in development of gut-associated lymphoid tissues and preimmune antibody repertorie. J Immunol (2004) 172:1118–24. 10.4049/jimmunol.172.2.1118 14707086

[37] Lee KW Li G Lillehoj HS Lee SH Jang SI Babu US *Bacillus subtilis*-based direct-fed microbials augment macrophage function in broiler chickens. Res Vet Sci (2011) 91:e87–91. 10.1016/j.rvsc.2011.01.018 21338997

[38] Molnár AK Podmaniczky B Kürti P Tenk I Glávits R Virág GY Effect of different concentrations of *Bacillus subtilis* on growth performance, carcase quality, gut microflora and immune response of broiler chickens. Br Poult Sci (2011) 6:658–65. 10.1080/00071668.2011.636029 22221231

[39] Hosoi T Ametani A Kiuchi K Kaminogawa S Improved growth and viability of lactobacilli in the presence of *Bacillus subtilis* (natto), catalase, or subtilisin. Can J Microbiol (2000) 46:892–7. 10.1139/cjm-46-10-892 11068675

[40] Jeong JS Kim IH. Effect of *Bacillus subtilis* C-3102 spores as a probiotic feed supplement on growth performance, noxious gas emission, and intestinal microflora in broilers. Poult Sci (2014) 93:3097–103. 10.3382/ps.2014-04086 25260523

[41] Okamoto K Fujiya M Nata T Ueno N Inaba Y Ishikawa C Competence and sporulation factor derived from *Bacillus subtilis* improves epithelial cell injury in intestinal inflammation via immunomodulation and cytoprotection. Int J Colorectal Dis (2012) 27:1039–46. 10.1007/s00384-012-1416-8 22297864

[42] Wolfenden R Pumford N Morgan M Shivaramaiah S Wolfenden A Tellez G Evaluation of a screening and selection method for *Bacillus* isolates for use as effective direct-fed microbials in commercial poultry. Int J Poult Sci (2010) 9:317–23. 10.3923/ijps.2010.317.323

[43] Jani SA Chudasama CJ Patel DB Bhatt PS Patel HN Optimization of extracellular protease production from alkali thermo tolerant actinomycetes: *Saccharomonospora viridis* SJ-21. Bull Environ Pharmacol Life Sci (2012) 1:84–92.

[44] Pailin T Kang DH Schmidt K Fung DYC. Detection of extracellular bound proteinase in EPS-producing lactic acid bacteria cultures on skim milk agar. Lett Appl Microbiol (2001) 33:45–9. 10.1046/j.1472-765X.2001.00954.x 11442814

[45] Nerurkar M Joshi M Adivarekar R Use of sesame oil cake for lipase production from a newly marine isolated *Bacillus sonorensis*. Innov Rom Food Biotechnol (2013) 13:11–7.

[46] Gulati HK Chadha BS Saini HS. Production, purification and characterization of thermostable phytase from thermophilic fungus *Thermomyces lanuginosus* TL-7. Acta Microbiol Immunol Hung (2007) 54:121–38. 10.1556/AMicr.54.2007.2.3 17899792

[47] Mittal A Singh G Goyal V Yadav A Aneja KR Gautam SK Isolation and biochemical characterization of acido-thermophilic extracellular phytase producting bacterial for potential application in poultry feed. Jundishapur J Microbiol (2011) 4:273–82.

[48] Lawson PA Citron DM Tyrrell KL Finegold SD Reclassification of *Clostridium difficile* as *Clostridioides difficile* (Hall and O’Toole 1935) Prevot 1938. Anaerobe (2016) 40:95–9. 10.1016/j.anaerobe.2016.06.008 27370902

[49] O’Toole GA Kolter R Initiation of biofilm formation in *Pseudomonas fluorescens* WCS365 proceeds via multiple, convergent signaling pathways: a genetic analysis. Mol Microbiol (1998) 28:449–61. 10.1046/j.1365-2958.1998.00797.x 9632250

[50] Fall R Kinsinger RF Wheeler KA. A simple method to isolate biofilm-forming *Bacillus subtilis* and related species from plant roots. Syst Appl Microbiol (2004) 27:372–9. 10.1078/0723-2020-00267 15214643

[51] Institute SAS. SAS User Guide. Version 9.1. Cary, NC: SAS Institute Inc (2002).

[52] Donohue M Cunningham DL Effects of grain and oil seed prices on the costs of US poultry production. J Appl Poult Res (2009) 18:325–37. 10.3382/japr.2008-00134

[53] Loar RE Moritz JS Donaldson JR Corzo A Effect of feeding distillers dried grains with solubles to broilers from 0 to 28 days posthatch on broiler performance, feed manufacturing efficiency, and selected intestinal characteristics. Poult Sci (2010) 89:2242–50. 10.3382/ps.2010-00894 20852115

[54] Barekatain MR Antipatis C Rodgers N Walkden-Brown SW Iji PA Choct M. Evaluation of high dietary inclusion of distillers dried grains with solubles and supplementation of protease and xylanase in the diets of broiler chickens under necrotic enteritis challenge. Poult Sci (2013) 92:1579–94. 10.3382/ps.2012-02786 23687155

[55] Kundsen KEB Carbohydrate and lignin contents of plant materials used in animal feeding. Anim Feed Sci Technol (1997) 63:319–38.

[56] Choct MR Hughes RJ Trimble RP Angkanapor K Annison G. Non-starch polysaccharide-degrading enzymes increase the performance of broiler chickens fed wheat of low apparent metabolizable energy. J Nutr (1995) 125:485–92. 7876924 10.1093/jn/125.3.485

[57] Avila E Arce J Soto C Rosas F Ceccantini M McIntyre DR Evaluation of an enzyme complex containing non-starch polysaccharide enzymes and phytase on the performance of broilers fed on a sorghum and soybean meal diet. J Appl Poult Res (2012) 21:279–86. 10.3382/japr.2011-00382

[58] Cowieson AJ Adeola O. Carbohydrases, protease, and phytase have an additive beneficial effect in nutritionally marginal diets for broiler chicks. Poult Sci (2005) 84:1860–7. 10.1093/ps/84.12.1860 16479942

[59] Ghazi S Rooke JA Galbraith H Improvement of the nutritive value of soybean meal by protease and α-galactosidase treatment in broiler cockerels and broiler chicks. Br Poult Sci (2003) 44:410–8. 10.1080/00071660310001598283 12964625

[60] Latorre JD Hernandez-Velasco X Bielke LR Vicente JL Wolfenden R Menconi A Evaluation of a *Bacillus* direct-fed microbial candidate on digesta viscosity, bacterial translocation, microbiota composition and bone mineralisation in broiler chickens fed on a rye-based diet. Br Poult Sci (2015) 56:723–32. 10.1080/00071668.2015.1101053 26539833

[61] Guo X Li D Lu W Piao X Chen X. Screening of *Bacillus* strains as potential probiotics and subsequent confirmation of the *in vivo* effectiveness of *Bacillus subtilis* MA139 in pigs. Antonie Van Leeuwenhoek (2006) 90:139–46. 10.1007/s10482-006-9067-9 16820971

[62] La Ragione RM Woodward MJ. Competitive exclusion by *Bacillus subtilis* spores of *Salmonella enterica* serotype Enteritidis and *Clostridium perfringens* in young chickens. Vet Microbiol (2003) 94:245–56. 10.1016/S0378-1135(03)00077-4 12814892

[63] Duc LH Hong HA Barbosa TM Henriques AO Cutting SM. Characterization of *Bacillus* probiotics available for human use. Appl Environ Microbiol (2004) 70:2161–71. 10.1128/AEM.70.4.2161-2171.2004 15066809 PMC383048

[64] Hong H Huang JM Khaneja R Hiep L Urdaci M Cutting S. The safety of *Bacillus subtilis* and *Bacillus indicus* as food probiotics. J Appl Microbiol (2008) 105:510–20. 10.1111/j.1365-2672.2008.03773.x 18312567

[65] Harvey RB Norman KN Andrews K Hume ME Scanlan CM Callaway TR *Clostridium difficile* in poultry and poultry meat. Foodborne Pathog Dis (2011) 8:1321–3. 10.1089/fpd.2011.0936 21877928

[66] Colenutt C Cutting SM. Use of *Bacillus subtilis* PXN21 spores for suppression of *Clostridium difficile* infection symptoms in a murine model. FEMS Microbiol Lett (2014) 358:154–61. 10.1111/1574-6968.12468 24828432

